# Lower cardiopulmonary resuscitation quality after pre-resuscitation physical exertion and stress exposure in active firefighters: a within-subject field study in a standardized operational setting

**DOI:** 10.1016/j.resplu.2026.101344

**Published:** 2026-04-24

**Authors:** Andrea Schittenhelm, Tom Brandt, Sean Paul Renker, Annette Schmidt

**Affiliations:** a dtec.bw, NextGenerationEU Project Smart Health Lab, University of the Bundeswehr, Chair of Sport Biology, Munich, Germany; b University of the Bundeswehr Munich, Institute of Sport Sciences, Chair of Sport Biology, Munich, Germany; c Research Center Smart Digital Health, University of the Bundeswehr, Munich, Germany; d Technical University of Munich, School of Medicine and Health, Department of Sport and Health Science, Sportpsychology Unit, Munich, Germany

**Keywords:** Emergency personnel, Fire brigade, Physical stress, Physiology

## Abstract

**Introduction:**

Cardiopulmonary resuscitation is a physically demanding intervention critical for survival in sudden cardiac arrest. While most studies focus on fatigue developing during resuscitation, emergency responders often initiate cardiopulmonary resuscitation after physically and psychologically demanding events. This study investigated changes in cardiopulmonary resuscitation performance following pre-exposure to physical and psychological stress in active firefighters.

**Methods:**

23 Caucasian firefighters (18 male, 5 female; body mass index = 25.4 ± 3.86; body fat = 19.7% ± 8.98) participated in a within-subject field design. Each participant performed a standardized two-minute cardiopulmonary resuscitation on a manikin before and after a fire service–typical operational task involving a self-contained breathing apparatus. Cardiopulmonary resuscitation performance was assessed using the SimPad Plus by Leardal Medical. Secondary outcomes included heart rate, salivary cortisol (nmol/L), alpha-amylase (U/mL), self-reported affect and anxiety.

**Results:**

Physiological markers indicated an acute stress response, with cortisol increasing by 236% (95% CI: 56–416%) and alpha-amylase by 60% (95% CI: 35–84%) (both *p* < 0.001). Cardiopulmonary resuscitation quality declined in overall (−24%, 95% CI: 12–36%, *p* = 0.002, *r* = 0.81) and compression scores (−36%, 95% CI: 18–55%, *p* = 0.001, *r* = 0.81).

**Discussion:**

These findings suggest that pre-resuscitation exertion may be associated with reduced CPR quality, which could be relevant for physically demanding emergency settings. They may therefore have implications for workload management and physical preparedness in emergency personnel.

## Introduction

In several Western countries, non-professional emergency service workers, such as volunteer firefighters, greatly contribute to citizens’ safety. Particularly in rural areas, they are the first to arrive in case of fires, environmental disasters, or other emergencies.^1^ Providing urgent care to casualties is, therefore, a vital aspect of their duty and is considered both physically and psychologically demanding.^2^ Especially, high-quality cardiopulmonary resuscitation (CPR) under highly stressful conditions can be seen as crucial medical treatment, given that, for example, 15–20% of all natural deaths in Western societies are due to sudden cardiac arrest.^3^ Since every second counts in such cases, instantly starting CPR is a key factor for survival. Besides timing, the American Heart Association mentions adequate compression depth (≥50 millimeters [mm] in adults) and rate (100–120/minute [min]) while minimizing pauses in compressions (80% of time should be spent with a chest compression) as cornerstones for successful CPR.^4^, ^5^

Concerning the physical demands, it is important to note that reaching the incident site can already be physically exhausting. Performing CPR represents an additional physical strain. This is critical as previous studies showed that within the first 2 min of CPR, the force applied during chest compression decreased by 50–60 Newton.^6^, ^7^ Furthermore, McDonald and colleagues found that 79% of study participants reported that fatigue negatively impacted their CPR performance.^8^ Again, half of the reduction in correct chest compressions occurred between the 1st and 2nd min of CPR. Considering that participants in the cited studies performed CPR whilst rested, the influence of pre-fatiguing tasks during real-life emergencies remains unclear, but could lead to pronounced adverse effects.

Besides physical stress, emergency responders face further psychological stressors like limited professional knowledge, unreasonable expectations, incompatibility with colleagues, sleep disturbance, and acute critical incidents (e.g., patient death, acute danger for emergency personnel).^9^, ^10^ According to a recent review by Vincent and colleagues,^11^ it is assumed that the above-mentioned psychological stressors could also decrease CPR performance. Furthermore, populations working under such conditions (e.g., firefighters, nurses, police officers, paramedics) are susceptible to posttraumatic stress symptoms as well as high levels of depression, anxiety, insomnia symptoms, fatigue, and narcolepsy, which could negatively affect cognitive function.^12^, ^13^

Given the detrimental effects of acute stress and physical exertion, preparing volunteer firefighters for task-related demands may improve resilience and performance under operational stress, with potential implications for physically demanding tasks such as CPR in emergency settings. Several previous studies support this notion. While Scofield and Kardouni emphasized the impact of effective training strategies in tactical populations in general for occupational performance, Hunter and colleagues suggested targeted training programs specifically for emergency responders.^2^, ^14^ However, to design specific, individualized training interventions for volunteer firefighters, it is essential to understand not only the objective external stressors (e.g., distance, load, intensity, duration, noise, temperature, hazardous substances, or acute danger) but also their impact on job-specific task performance and the physiological and psychological responses they elicit. While the aforementioned studies provide valuable insights, no investigations to date have combined these measurements within the context of a comprehensive, job-specific task for volunteer firefighters.

In the present study, we investigated changes in CPR performance following pre-exposure to physical and psychological stress in active volunteer firefighters. To induce stress, we used the standardized annual self-contained breathing apparatus (SCBA) test of the German fire brigade, which provided a practical and controlled opportunity to elicit multidimensional stress.

## Methods

### Participants

Participants were active volunteer firefighters aged ≥18 years, certified to wear SCBA, and at least trained as first responders. Individuals were excluded if they were medically unfit to participate in SCBA training, failed to complete the SCBA test, or used stimulants (caffeine, nicotine) up to 30 min before testing. We recruited from local fire brigades. We contacted fire departments directly or by mail to identify scheduled SCBA training sessions during the study period. Data collection was conducted during these sessions with approval from the respective training supervisors. We did not formally assess physical fitness; however, all participants had successfully completed SCBA re-certification, which requires a level of physical fitness sufficient for operational tasks. Experience with SCBA use varied by certification duration, although all participants were familiar with the SCBA certification procedure (for more details, see Results).

### Trial oversight

This study employed a single-arm intervention design with pre- and post-intervention measurements to assess CPR performance and physiological and psychological stress responses. All measurements and the SCBA intervention were conducted at two fire departments in Bavaria, Germany. Test personnel and participants were not blinded. A physician was always on-site and observed the participants' heart rate (HR) throughout the intervention to minimize the risk of adverse events. Personnel of the participating fire departments ensured compliance with standardized procedures and conditions during the SCBA intervention.

Although SCBA-certified firefighters do not typically perform CPR, the SCBA exercise was selected because it represents a standardized task that combines substantial physical strain with performance-related psychological stressors (e.g., time pressure and task demands), thereby providing greater ecological validity than laboratory-based stress paradigms. While the addition of CPR does not replicate real-life emergency scenarios, the exercise captures key aspects of operational stress under physically demanding conditions. We were particularly interested in how such stressors, commonly encountered by emergency personnel (e.g., paramedics), affect CPR performance. In addition, the standardized and supervised nature of the assessment ensured participant safety and procedural consistency.

**Fig. 1:**
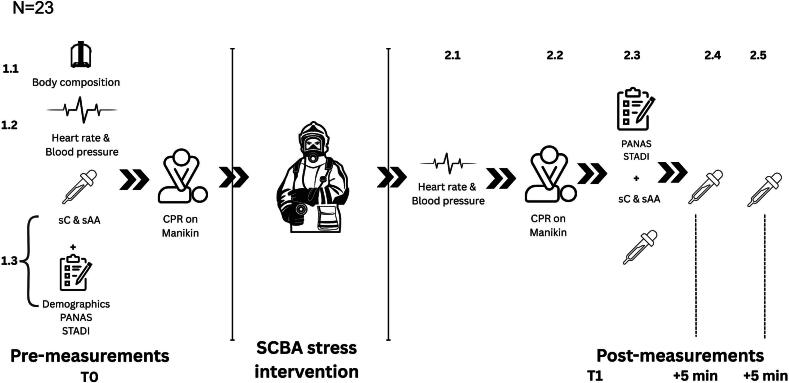
**Within-subject study design and measurement schedule**. Numbers (#1–5) indicate test order. **Abbreviations:** see text. Illustration was made in Canva Pro.

The Institutional Ethics Committee of the University of the Bundeswehr Munich (01/10/2024; EK UniBw M 25-04) approved the study protocol, confirming that it complied with the ethical guidelines of the 1975 Declaration of Helsinki. Informed consent was obtained from all subjects involved in the study. The data protection concept for this study was reviewed and approved by the university’s data protection officer before data collection. The study design is displayed in Fig. 1. All data can be obtained upon request.

### Protocol and outcomes

All tests took place in the late afternoon and early evening. No morning saliva samples were collected. Before the test session, participants were introduced to the study and consented to participate. They were further asked if they had any health issues or consumed medications that could prevent them from attending the testing or intervention. Additionally, a questionnaire was used to verify whether participants had refrained from intense physical activity within the 24 h before testing, as well as from caffeine and nicotine consumption in the hour leading up to it. Then, demographic data, body composition, as well as resting HR and blood pressure were assessed. To capture the psychological correlates of the stress response, we assessed affective state using the PANAS^15^ as well as subscales of anxiety and concern of the STADI.^16^ These instruments index affective and anxiety responses and are treated as correlates of stress. To estimate the relative rate of perceived exhaustion, the Borg scale was used.^17^ Saliva samples were obtained to measure the physiological stress response via cortisol [nmol/l] and alpha-amylase [U/ml] during rest. For the pre-measurement of CPR performance, participants put on their uniform and gear (12 kg), not the SCBA apparatus (10 kg). They were then informed that an unconscious adult had been found on the premises and that they should examine them according to first-aid guidelines (addressing pain stimulus, pulse check, breathing check) and, if no circulation was detected, initiate 2-min CPR on the Resusci Anne V (Laerdal Medical GmbH, Puchheim, Germany). CPR was always performed by a single rescuer to assess the effects of pre-resuscitation exertion on individual CPR performance, without the influence of team-based fatigue management strategies. After that, participants proceeded with the SCBA testing, which all volunteer firefighters must complete annually. The SCBA followed the standardized test protocol according to the German Fire Brigade service regulations. As part of the procedure, participants donned the SCBA equipment, resulting in a total load of 22 kg. To pass the test, participants must complete physical tasks that amount to a workload of 80 kJ using a maximum of 1600 L of air supplied by the SCBA apparatus. The physical tasks included climbing a ladder, using a bicycle ergometer, using a treadmill, using an arm ergometer, and completing an orientation course (a dark, noisy, and foggy tunnel with a slight height difference).

After completing the SCBA test, participants removed their SCBA equipment, after which HR and blood pressure were assessed. Subsequently, they received the same verbal instruction regarding the unconscious person and performed two minutes of CPR on Resusci Anne V. Following the CPR task, post-assessments of rate of perceived exhaustion, PANAS, and STADI were conducted. In addition, saliva samples were collected immediately after CPR and five and ten minutes later.

The primary outcome was the change in CPR performance measures from pre- to post-intervention. Secondary outcomes included changes in physiological and psychological stress parameters between pre- and post-measurement. For exploratory purposes, we conducted correlations between CPR performance and stress parameters (physiological and psychological) and linear regressions.

#### Body composition

Body composition was measured using a bioelectrical impedance analysis device (Tanita BC-545N, Tanita Europe B.V., Stuttgart, Germany).

#### Heart rate and blood pressure

HR and blood pressure were measured with the electric blood pressure monitor Carat Professional (BOSCH + SOHN GmbH & Co. KG, Germany).

#### Cardiopulmonary resuscitation performance

The CPR testing was conducted with a Resusci-Anne V (Laerdal Medical GmbH, Puchheim, Germany). The procedure included the assessment of vital signs (responsiveness, breathing, pulse, etc.), and resuscitation in a 30:2 rhythm for 150 s. The resuscitation data were recorded with a SimPad Plus (Laerdal Medical GmbH, Puchheim, Germany). The CPR was evaluated based on the compression, ventilation, and overall scores (see Table 2).

#### Positive and negative affect scale

The German version of the PANAS^15^ is used to assess the participants' emotional state. The PANAS comprises 20 adjectives describing various sensations and emotions, with 10 adjectives representing positive affect and 10 representing negative affect. The participants rated each adjective on a scale ranging from 1 to 5 (1 = not at all, 2 = a little, 3 = moderately, 4 = quite a bit, 5 = extremely). The mean for each dimension was calculated based on the 10 adjectives, with higher scores indicating greater positive affect (PA) or negative affect (NA).

#### State-trait anxiety depression inventory

The German version of the State-Trait Anxiety Depression Inventory (STADI) was used to measure participants' state anxiety based on the subscales of arousal (affective component) and worry (cognitive component). Each subscale has five items rated by the test subjects on a scale of 1–4 (1 = not at all; 2 = a little, 3 = quite a bit 4 = very much). Examples of items for excitement are: “I feel jittery” or “my heart is beating fast.” Examples of items for concern are: “I worry about what lies ahead,” or “I am unsure whether everything will go well.” The sum of the subscale values for excitement and concern yields the state anxiety value.

#### Relative perceived exertion

As a subjective measure of physical effort, the relative perceived exertion was assessed using the Borg scale, which ranges from 6 (lowest exertion) to 20 (highest exertion), aligning with the HR scale for exercise intensity.

#### Salivary cortisol and alpha-amylase

Saliva samples were collected via salivettes (Sarstedt, Nümbrecht, Germany). Therefore, participants held the salivette in their mouth for 120 s, moving it side to side without chewing. Saliva samples were stored and analyzed with the same protocol as Fellinger and colleagues.^18^

### Statistical analysis

We used Excel (Microsoft, 2019) and JASP Version 0.16.4 (JASP Team, 2022) for statistical analysis. No cortisol or alpha-amylase values were obtained for two participants due to insufficient saliva volume. Four and five participants, respectively, showed higher pre-intervention than post-intervention cortisol and alpha-amylase levels. Since not all dependent variables were normally distributed (Shapiro–Wilk, *p* < 0.05) and the sample size was small, the Wilcoxon signed-rank test was used to assess pre-post intervention differences. For the exploratory analysis, we used Spearman correlation.

## Results

### Participants

The study involved healthy adults (*N* = 23, 78% male, 22% female). The average age was 28.8 years ± 8.86, ranging from 19 to 47 years. The average body mass index was 25.4 kg/m^2^ ± 3.86 at an average body fat percentage of 19.7% ± 8.98. While all participants served in various fire brigades across Munich, Bavaria, Germany, they differed according to their levels of qualification. Regarding medical qualification level, *n* = 8 participants were first responders, *n* = 9 had first responder and paramedic qualifications, *n* = 4 were emergency medical technicians (EMT), and *n* = 2 were trained as medical professionals. Among the participants, *n* = 5 reported being certified as SCBA wearers for less than one year, *n* = 5 for 2–5 years, *n* = 4 for 6–10 years, and *n* = 9 for more than 10 years. The demographic survey revealed no influence of medication, illnesses, unusual sleeping patterns, or consumption of stimulants for any participant. Table 1 provides a detailed overview of descriptive statistics for demographics.

**Table 1:** Descriptive statistics for demographics of the participants (mean, 95% confidence interval, standard deviation).

**Variable**	**Mean**	**95% CI upper**	**95% CI lower**	**Std. deviation**
Age	28.783	32.615	24.950	8.862
Years of service	3.304	3.635	2.974	0.765
BMI	25.391	27.060	23.723	3.858
Body fat (%)	19.696	23.580	15.812	8.982

### Descriptive statistics

Table 2 lists all variables obtained.

**Table 2:** Changes between pre- and post-intervention values (mean differences, 95% confidence intervals, *p*-values, and effect sizes, Wilcoxon tests).

**Variable**	**Mean change (*Δ*)**	**95% CI**	***p***	**ES (*r*)**
Overall CPR score (%)	−14.62	−22.10 to −7.14	0.002	0.814
Compression score (%)	−26.00	−39.24 to −12.76	0.001	0.810
Deep enough compressions (%)	−9.10	−25.52 to 7.33	0.205	0.337
Compressions with adequate rate (%)	−11.76	−33.06 to 9.54	0.251	0.305
Average compression rate (cpm)	−41.29	−87.08 to 4.50	0.737	−0.090
Average compression depth (mm)	−90.48	−404.83 to 223.87	0.442	0.195
Ventilation score (%)	−1.62	−11.61 to 8.37	0.587	0.147
Ventilations with adequate volume (%)	−3.48	−13.23 to 6.28	0.601	0.146
Average ventilation volume (mL)	137.43	−4.63 to 279.48	0.067	−0.484
Ventilations below minimum volume (%)	8.81	−4.49 to 22.10	0.234	−0.385
Ventilations above maximum volume (%)	4.48	−8.99 to 17.95	0.717	−0.100
Heart rate (beats/min)	34.30	25.11 to 43.50	<0.001	−0.988
Positive affect (points)	3.96	0.64 to 7.27	0.005	−0.680
Negative affect (points)	−2.30	−3.95 to −0.66	0.012	0.678
State anxiety (points)	−0.09	−1.99 to 1.81	0.903	−0.035
Excitement (sum-points)	1.26	0.21 to 2.31	0.023	−0.581
Concern (sum-points)	−1.35	−2.45 to −0.24	0.018	0.724
Salivary cortisol (nmol/L)	5.18	1.23 to 9.13	<0.001	−1.000
Salivary alpha-amylase (U/mL)	65.62	39.06 to 92.18	<0.001	−1.000

### Primary outcome

**Fig. 2:**
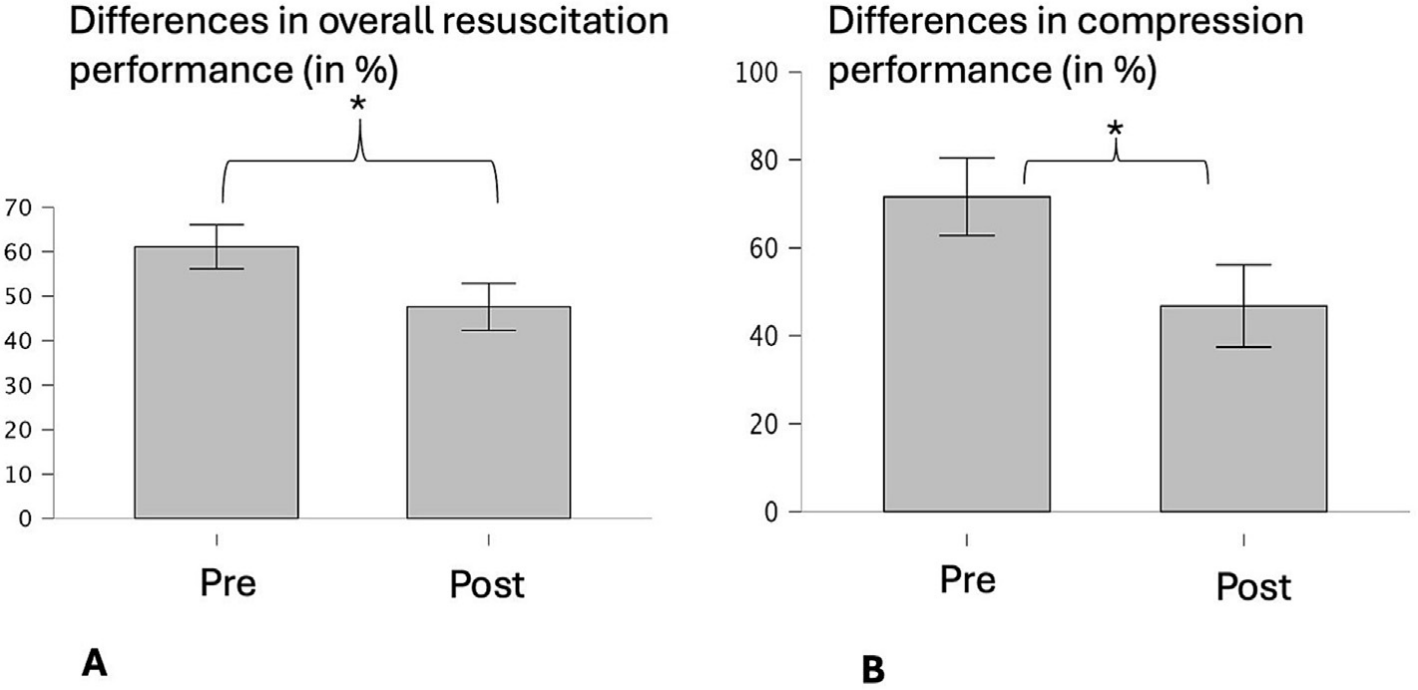
**Differences in CPR variables between pre- and post-measurements. Values are mean values in percent. Error bars correspond to the standard deviation**. (A) Differences in overall CPR score (%) pre and post SCBA intervention, (B) Differences in compression performance (%) pre and post SCBA intervention.

After the SCBA intervention, we saw a significant decline in overall resuscitation performance (*z* = 3.19; *p* = 0.002; *r* = 0.81) (−22%) and the compression score (*z* = 3.25; *p* = 0.001; *r* = 0.81) (−35%). See Fig. 2. Other compression-related parameters, as well as ventilation-related measures, did not show significant changes (all *p* > 0.05).

### Secondary outcomes

Cortisol and alpha-amylase increased significantly between the baseline and peak post-samples. Cortisol values increased on average by ∼236% (*z* = −3.52; *p* < 0.001; *r* = −1.00) and sAA by ∼60% (*z* = −3.52; *p* < 0.001; *r* = −1.00). The HR increased significantly (*z* = −4.058; *p* < 0.001, *r* = −0.988). Anxiety did not differ significantly (*z* = −0.139; *p* = 0.903, *r* = −0.035). Additionally, we calculated whether the anxiety subscales differed pre-post. Excitement (*z* = −2.28; *p* = 0.023, *r* = 0.58) increased significantly. Whereas concern (*z* = 2.39; *p* = 0.018, *r* = 0.72) decreased. In line with these results, PA (*z* = −2.79; *p* = 0.005, *r* = −0.68) increased significantly. Meanwhile, NA (z = 2.53; *p* = 0.012, *r* = 0.68) decreased significantly.

### Explorative outcomes

We did an additional exploratory correlation analysis that included changes (*Δ*) in psychological, physiological, and performance variables. The focus was to determine whether physiological or psychological markers are associated with performance markers. Hence, correlations within performance markers (e.g., compression and ventilation) and psychological markers (e.g., positive affect and anxiety) were neglected. A significant negative correlation was observed between *Δ* compression score and *Δ* negative affect (*r* = −0.37, *p* = 0.05), between *Δ* compression score and *Δ* excitement (*r* = −0.39, *p* = 0.04), and *Δ* between ventilations above maximum volume and *Δ* excitement (*r* = −0.43, *p* = 0.03). No other correlations were statistically significant. We further conducted exploratory linear regression analyses for the significant metrics, overall score and compression score, to examine whether changes in physiological stress markers (*Δ* cortisol, *Δ* alpha-amylase) were associated with post-intervention CPR performance, controlling for baseline performance. For overall CPR quality, the model was statistically significant (*R*^2^ = 0.65, *F*(3,9) = 5.60, *p* = 0.019). However, this effect was primarily driven by baseline performance, with overall score-pre emerging as a significant predictor (*β* = 0.86, *p* = 0.009). In contrast, neither *Δ* cortisol (*β* = 0.07, *p* = 0.781) nor *Δ* alpha-amylase (*β* = 0.08, *p* = 0.683) were significantly associated with overall CPR performance. For compression score, the model was not statistically significant (*R*^2^ = 0.29, *F*(3,9) = 1.21, *p* = 0.361). None of the predictors, including baseline compression score (*β* = 0.24, *p* = 0.432), *Δ* cortisol (*β* = −0.47, *p* = 0.135), or *Δ* alpha-amylase (*β* = −0.02, *p* = 0.960), showed significant associations with post-intervention compression performance. None of the psychological predictors was a significant predictor of CPR performance.

## Discussion

In contrast to many previous studies that focused on fatigue resulting from CPR itself, we examined changes in CPR quality following pre-resuscitation physical exertion and stress-related psychological responses. To enhance ecological validity relative to laboratory-based studies, we selected a physically and mentally demanding, operationally relevant intervention. Our approach used a multidimensional assessment of stress, incorporating biological, physiological, and psychological measures. Primary outcomes were CPR quality, while secondary outcomes included biomarker reactivity and self-reported affect and anxiety. In contrast to Abraldes et al.,^19^ who reported a slight improvement in compression quality following physical exertion, our results showed a significant decline in compression scores, suggesting that combined physical and psychological load may compromise CPR performance. In line with this, Gianotto-Oliveira et al.^20^ demonstrated that prolonged CPR reduces compression quality. Together, these findings indicate that physical fatigue, whether developing during or prior to CPR, may impair compression performance from the outset. In our study, CPR quality declined following the SCBA intervention, reflected by reduced compression scores. This may be relevant for real-world emergencies, where pre-fatigue could limit the ability to maintain adequate compression quality. Previous studies suggest potential strategies to mitigate fatigue-related declines in CPR quality. Short breaks during CPR have been shown to improve compression quality and reduce rescuer fatigue,^21^ while higher physical fitness is associated with better CPR performance.^22^ These findings highlight the potential value of incorporating rest strategies and physical conditioning into CPR training.

Physiological and endocrine markers indicated that the SCBA intervention elicited an acute stress response, as both cortisol and alpha-amylase increased from baseline to peak post-intervention. However, these physiological markers were not associated with CPR performance in either correlation or regression analyses. These findings should be interpreted with caution, given the exploratory nature of the analyses and the small sample size.

In contrast to Vincent et al.,^11^ who emphasized psychological factors over biological stress markers, our findings suggest a different pattern. Impairments in CPR performance coincided with marked increases in biological stress indicators, particularly salivary cortisol, while psychological measures indicated a more positive stress appraisal, with positive affect and excitement increased, whereas concern and negative affect decreased. This may suggest that reduced CPR quality could be more closely related to physical exertion than to psychological strain, indicating elevated arousal without substantial emotional distress. This pattern is consistent with the concept of challenge appraisal as described by Lazarus and Folkman,^23^ where stress is associated with adaptive activation rather than impairment. Taken together, these findings may suggest that the physically demanding nature of the SCBA exercise may have had a stronger impact on CPR performance than psychological stress.

Several limitations should be considered when interpreting the present findings. First, although the SCBA exercise provides a standardized, multidimensional stress paradigm that captures key aspects of operational stress, the combination of SCBA exposure and subsequent CPR does not reflect routine clinical practice, which may limit the direct transferability of the findings. Second, the sample size was relatively small (*N* = 23). Third, participants’ physical fitness was not formally assessed. Fourth, the absence of a control condition prevents clear attribution of the observed decline to specific elements of the protocol, such as the passage of time, the initial CPR bout, the SCBA exercise, or their combined effects. Finally, the multifactorial nature of the SCBA intervention precludes disentangling the relative contributions of physical and psychological stress components.

## Conclusion and further implications

Our data may suggest that acute pre-resuscitation exertion and stress-related psychophysiological responses are associated with lower CPR quality. While the study scenario does not directly reflect routine clinical practice, it captures relevant aspects of operational stress that may also occur in emergency settings. These findings may therefore have implications for workload management among emergency personnel, particularly in minimizing pre-resuscitation fatigue and ensuring adequate physical readiness before initiating CPR. In addition, although not examined in the present study, physical fitness may represent a relevant factor in mitigating fatigue-related performance declines and warrants further investigation.

## Institutional review board statement

The study was conducted in accordance with the Declaration of Helsinki and approved by the Institutional Review Board of the University of the Bundeswehr Munich for studies involving humans.

## Informed consent statement

Informed consent was obtained from all subjects involved in the study. Further, written informed consent has been obtained from the patients to publish this paper.

## Declaration of use of AI-based tools

The authors used the following AI-Tools. After using the tools, the authors reviewed and edited the content as needed and take full responsibility for the content of the published article:

**Table:** 

AI-based tool	Use case	Scope	Remarks
ChatGPT 4.0	Creation of tables; structural guidance for sections; simplification/clarification of sentences; creation of simple icons for the study overview	Selected sections: Discussion, abstract, study-overview figure, tables	All outputs were reviewed and edited by the authors; no confidential data were entered
ChatGPT 5	Same as above; iterative refinement of wording and structure in later drafts		
DeepL Pro	Translation of text passages	Entire work	From German to English
Grammarly	Checking and correcting grammar, spelling, punctuation, and sentence structure	Entire work	Premium Plan; integrated in Microsoft Word

## CRediT authorship contribution statement

**Andrea Schittenhelm:** Writing – review & editing, Writing – original draft, Methodology, Formal analysis, Conceptualization. **Tom Brandt:** Writing – review & editing, Supervision, Formal analysis. **Sean Paul Renker:** Conceptualization. **Annette Schmidt:** Writing – review & editing, Supervision, Methodology, Funding acquisition, Conceptualization.

## Funding

The Center for Digitization and Technology Research of the German Bundeswehr funded this research and the associated Smart Health Lab (SHL) project. Dtec.bw is funded by the European Union, NextGenerationEU.

## Declaration of competing interest

The authors declare no conflict of interest.
